# A necroptosis-related lncRNA signature was identified to predict the prognosis and immune microenvironment of IDH-wild-type GBM

**DOI:** 10.3389/fonc.2022.1024208

**Published:** 2022-12-19

**Authors:** Chong Song, Liwen Zhu, Junwei Gu, Tong Wang, Linyong Shi, Chiyang Li, Lei Chen, Sidi Xie, Yuntao Lu

**Affiliations:** ^1^ Department of Neurosurgery, Nanfang Hospital, Southern Medical University, Guangzhou, China; ^2^ Department of Neurosurgery, The Central Hospital of Dalian University of Technology, Dalian, China; ^3^ Nanfang Neurology Research Institution, Nanfang Hospital, Southern Medical University, Guangzhou, China; ^4^ Nanfang Glioma Center, Nanfang Hospital, Southern Medical University, Guangzhou, China

**Keywords:** necroptosis, glioblastoma, tumor microenvironment, signature, prognosis

## Abstract

**Introduction:**

Necroptosis-related genes are essential for the advancement of IDH-wild-type GBM. However, the putative effects of necroptosis-related lncRNAs (nrlncRNAs) in IDH-wild-type GBM remain unknown.

**Methods:**

By using the TCGA and GTEx databases, a nrlncRNA prognostic signature was created using LASSO Cox regression. The median risk score was used to categorize the patients into low and high-risk groups. To confirm the validity, univariate, multivariate Cox regression and ROC curves were used. Furthermore, by enrichment analysis, immune correlation analysis, and drug sensitivity analysis, the targeted lncRNAs were selected for further verification. As the highest upregulated expression in tumor than peritumor specimens, RP11-131L12.4 was selected for phenotype and functional experiments in primary GBM cells.

**Results:**

Six lncRNAs were proved to be closely related to necroptosis in IDH-1-wild-type GBM, which were used to create a new signature. For 1-, 2-, and 3-year OS, the AUCs were 0.709, 0.645 and 0.694, respectively. Patients in the low-risk group had a better prognosis, stronger immune function activity, and more immune cell infiltration. In contrast, enrichment analysis revealed that the malignant phenotype was more prevalent in the high-risk group. In vitro experiments indicated that RP11-131L12.4 increased the tumor proliferation, migration and invasion, but decreased the necroptosis. Moreover, this nrlncRNA was also proved to be negatively associated with patient prognosis.

**Conclusion:**

The signature of nrlncRNAs may aid in the formulation of tailored and precise treatment for individuals with IDH-wild-type GBM. RP11-131L12.4 may play indispensable role in necroptosis suppression.

## Introduction

GBM is the most lethal CNS tumor in adults, and it has strong heterogeneity (1). According to the 2021 WHO Classification of Tumors of CNS, GBM, integrates three genomic factors as diagnostic criteria for IDH-wild-type GBM. As a result, adults with IDH-wild-type diffuse and astrocytic gliomas should be diagnosed with IDH-wild-type GBM if there is microvascular proliferation or necrosis, TERT promoter mutation, EGFR gene amplification, or +7/10 chromosome copy number alterations (2, 3). However, patients with the same molecular type still have a large difference in prognosis, and the effects of radiotherapy, chemotherapy and immunotherapy also differ (4, 5). This shows that some modest elements continue to influence prognosis and treatment response.

Necroptosis is a type of programmed necrotic cell death that can recognize pathogens and promote tissue repair (6). Some studies have found that NRGs have a role in a variety of tumor-related activities, however they appear to be a double-edged sword (7, 8).MLKL,RIPK1 and RIPK3 are the key mediators among them (9). RIPK1 and RIPK3 activation can alter associated signaling pathways to modulate the TME and perform a beneficial effect in anticancer progression (10–12). MLKL activation, on the other hand, is linked to highly aggressive tumor behavior and an immunosuppressive microenvironment (13, 14). Moreover, tumor cells can increase metastasis and extravasation by inducing necroptosis of the epithelial microvasculature (15). Therefore, the occurrence of necrotic apoptosis in tumors and its effect on tumor cells are very complex and worth further study.

LncRNAs are a type of noncoding RNA that has a length of more than 200nt and is implicated in the growth and metastasis of GBM. They play a crucial role in transcriptional suppression, transcriptional activation, chromosomal remodeling, and nuclear transport (16–18). LncRNAs are also vital in mediating necroptosis. For instance, it has been reported that the lncRNA H19-derived microRNA-675 could decrease the expression of FADD and enhance the necroptosis of HCC (19). Furthermore, the lncRNA HABON showed a protective effect on HCC cells under hypoxia by inhibiting mPTP opening (20). However, there have been few investigations on nrlncRNAs in GBM. The predictive usefulness of nrlncRNAs in GBM and its association with the TME remain unknown.

Therefore, in order to investigate the prognostic significance and prospective therapeutic options of nrlncRNAs in IDH-wild-type GBM and to elucidate the role of nrlncRNAs in the TME, the following research was conducted: we developed a predictive risk model based on nrlncRNA to predict the prognosis of IDH-wild-type GBM patients and serve as a guide for clinical diagnosis and treatment.

## Materials and methods

### Ethics statement

The Institutional Review Board at Nanfang Hospital of Southern Medical University provided written authorization and ethical approval for the use of human brain tumor specimens and the database (Guangzhou, China).

### Data download and processing

To determine deNRGs and delncRNAs at the transcript level, HTSeq-FPKM RNA sequencing profiles linked with primary GBM and normal brain tissues were collected from the TCGA database and the GTEx project. Initially, there were a total of 144 tumors and 1152 controls. Additionally, available clinical information of patients diagnosed with GBM, including age, sex and survival, was retrieved. ID conversion between transcripts (UCSC) and gene symbols was performed with the annotation file “gencode.v38.annotation.gtf”. If multiple transcripts represented the same gene, their median was used; if one transcript represented multiple genes, this transcript was deleted.

### Patient selection

The following inclusion criteria for patient enrollment were specified in order to examine nrlncRNAs and construct a prognosis prediction model in patients with IDH-wild-type GBM (1): patients were diagnosed with primary GBM with wild-type IDH; and (2) living status (yes/no) and OS were available. Consequently, 128 patients were selected for the following analyses (**Figure S1**), and their clinical characteristics are described in **Table S1**. To develop a prediction model for survival outcome, the GBM patients were randomly divided into training and testing datasets at a ratio of 2:1.

### Selection of NRGs and lncRNAs

Herein, genes involved in the KEGG pathway “Necroptosis” (hsa04217) from the NRG set and a total of 159 NRGs were retrieved through the R package “KEGGREST”.

### Identification of differentially expressed NRGs and lncRNAs

RNA sequencing data in FPKM values represent the intensity of transcripts on a log-2 scale. To identify deNRGs and delncRNAs between GBM and normal controls, a moderated t-statistic was carried out with the R package “limma”. Both the adjusted P value (Pa, Benjamini & Hochberg) and FC were obtained, and only those with Pa< 0.05 and |log2FC| > 1.0 were selected as a deNRG or delncRNA.

### Identification of OS-associated NRGs and lncRNAs

Patients with GBM were subsequently separated into two groups with expression higher or lower than the median for each deNRG and delncRNA, followed by a univariate Cox PH model. The deNRGs and delncRNAs with P<0.1 were regarded as OS-associated NRGs and lncRNAs.

### Identification of necroptosis-related lncRNAs

Pearson’s correlation analyses were performed to identify lncRNAs significantly correlated with OS-associated NRGs with both Pa<0.05 (Benjamini & Hochberg) and correlation coefficient |r| >0.3. Then, lncRNAs that were (1) significantly correlated with OS-associated NRGs and (2) included in the OS-associated lncRNAs were regarded as necroptosis-related lncRNAs and selected for developing the nrlncRNA signature as well as the prediction model.

### Necroptosis-related lncRNA signature construction and risk score calculation

In the training dataset, a nrlncRNA signature was created and subsequently verified in the testing dataset. That is, a multivariate Cox PH model with LASSO for variable selection and 10-fold cross-validation was run on necroptosis-related lncRNAs as continuous variables using the R package “glmnet”. The lncRNAs that had a nonzero coefficient in the regression finally formed the necroptosis-related lncRNA signature. Then, as shown in Equation, each patient was given a RS, which was a linear mixture of the independent prognostic indicators (expression of lncRNAs) weighted by their Cox regression coefficients. Differences in RSs among subgroups of patients with different ages and sexes were examined by Wilcoxon tests and Kruskal-Wallis tests. Additionally, subgroups of patients at low and high risk were defined based on the median RS. The Kaplan-Meier method with the log-rank test (R package “survminer”) was used to generate survival curves. The necroptosis-related lncRNA signature was used to generate a heatmap (R package “pheatmap”). We calculated the RS with the following formula:


$$Score=\sum_{i=0}^{n}\beta i*Xi$$

### Prediction model construction

To develop a prediction model for survival outcome, the GBM patients were randomly assigned to training and testing datasets in a 2:1 ratio. A multivariate Cox PH model with the RS and clinical characteristics was developed in the training dataset, and this prediction model was externally validated in the testing dataset. A nomogram was built based on the model to graphically forecast the 1-, 2-, and 3-year OS probabilities, and calibration curves were created to demonstrate the nomogram’s goodness of fit.

### Possible functions related to the necroptosis-related lncRNA signature

To determine the potential roles of the necroptosis-related lncRNA signature, patients were separated into low- and high-risk groups based on the median RS, and differential expression studies were performed. Then, utilizing the well-known GO and KEGG databases, functional enrichment analysis was done with the R tool “clusterProfiler”. Differentially expressed genes were annotated using BP, MF, and CC keywords, as well as KEGG pathways. With Pa<0.05 (Benjamini & Hochberg), GO keywords and KEGG pathways were deemed substantially enriched. Furthermore, GSVA was carried out to identify signature gene sets that reflect distinct well-defined biological states or processes (Pa<0.05 determined using the Benjamini and Hochberg technique).

### Evaluation of immune cell infiltration

The immune infiltration statuses in tumors were evaluated using the findings of functional enrichment analysis and GSVA. To compute the immune cell compositions for each sample,the analytical tools CIBERSORT, XCELL, ssGSEA, EPIC, MCP-counter, and QUANTISEQ were used. Additionally, another analytical tool, “ESTIMATE”, was utilized to evaluate immune cell infiltration (immune score), the presence of stroma (stromal score), and tumor purity (ESTIMATE score). The expression of 20 immunological checkpoint genes that might be targeted, as shown in **Table S2** [PMID: 26420858], were retrieved. The Wilcoxon test was used to examine differences in these metrics, which included immune cell signature compositions, immunological score, stromal score, ESTIMATE score, and immune checkpoint gene expression, across subgroups of patients with high and low RSs.

### Evaluation of drug sensitivity

Information from the Genomics of GDSC database, which describes 1000 human cancer cell lines and hundreds of chemicals, was utilized to assess the treatment sensitivity of GBM. The IC50 for each GBM patient was calculated using RNA sequencing data. The IC50 value was then examined using the Wilcoxon test across subgroups of individuals with high and low RSs, and its association with RS was assessed using Pearson’s correlation analysis.

### RNA isolation and real-time qRT−PCR

The levels of mRNA expression were determined using the qRT-PCR, as previously reported (21). The levels of mRNA expression were standardized to those of GAPDH. The **Supplemental Materials and Methods**, which are available online, provide a full list of primers.

### Cell culture and transfection

We obtained IDH-wild-type GBM primary cells for cultivation and transient knockdown of RP11-131L12.4. The **Supplemental Materials and Methods**, which are available online, provide a complete list of antibodies.

### Western blotting and antibodies

Western blotting was carried out as previously reported (21). The loading control was GAPDH. The **Supplemental Materials and Methods**, which are available online, provide a complete list of antibodies.

### Cell viability assay

The vitality of cells was determined using the CCK-8 and colony-forming assays. The **Supplemental Materials and Methods**, which are available online, provide a complete list of reagents.

### Cell migration and invasion assays

Wound healing and Transwell assays were used to measure cell migration and invasion. The **Supplemental Materials and Methods**, which are available online, provide a complete list of reagents.

### Immunohistochemical staining

Tissue section staining was performed as previously described (22), and the details of the staining and the scoring system for determining the percentage of positive cells and staining intensity are available in the **Supplemental Materials and Methods**.

### Statistical analysis

The analyses were carried out using the R programming environment (version 4.1.1) and GraphPad Prism 8.2.1. (GraphPad Software, San Diego, USA). See **Supplemental Materials and Methods** available online for details.

## Results

### Necroptosis-related lncRNAs in patients with IDH-wild-type GBM

**Figure 1:**
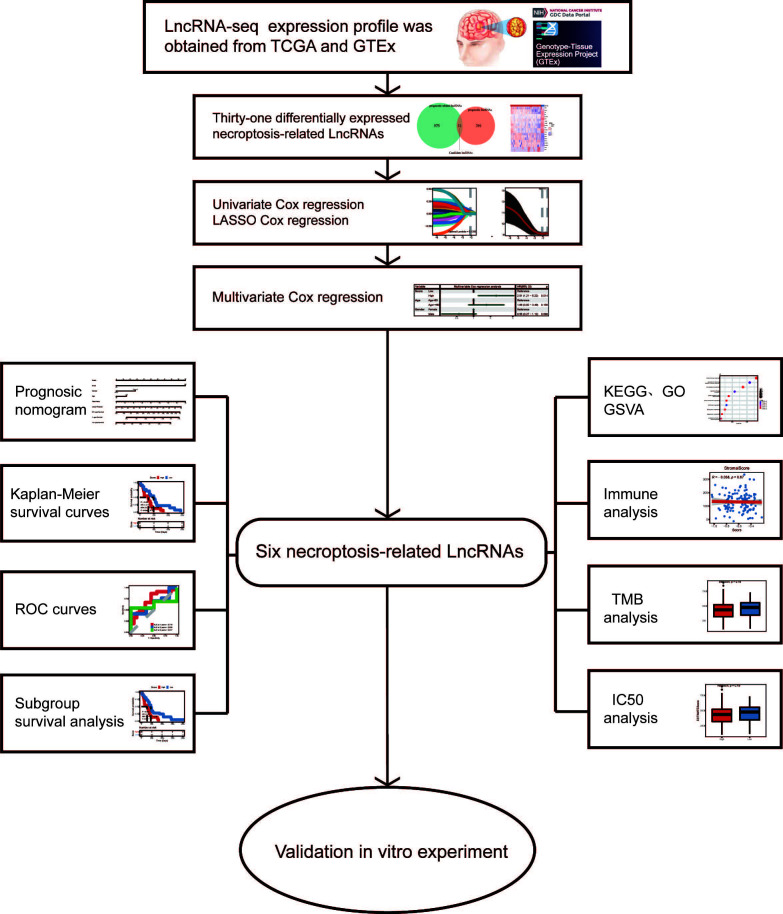
Design flow diagram for the research.

**Figure 2:**
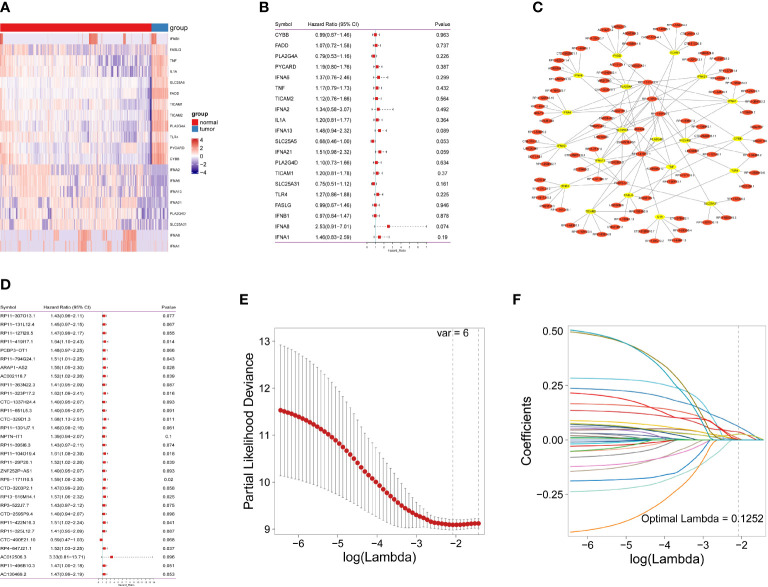
Identification of necroptosis-related lncRNAs in IDH-wild-type GBM patients. **(A)** Necroptosis-related gene expression in heatmap. **(B)** The predictive value of 20 necroptosis-related genes is depicted as a forest plot. **(C)** A network of differentially expressed genes and necroptosis-related lncRNAs. **(D)** A forest plot depicting the predictive significance of 31 necroptosis-related lncRNAs. **(E)** The vertical black line in the figure indicates the best logλ value. **(F)** Necroptosis-related lncRNA LASSO coefficient profile; each line represents an individual lncRNA.

As shown in **Figure 1** and **Figure S1**, the data of 434 patients with IDH-wild-type GBM were initially retrieved from TCGA; then, a total of 128 patients with RNA sequencing data and complete survival information remained for the following analyses (**Table S1**). In addition, 1152 normal brain tissues were obtained as controls from GTEx. Aberrant transcriptional profiles were examined between cases and controls; consequently, 20 out of 159 NRGs were significantly differentially expressed (|log2FC| > 1.0 and Pa< 0.05, **Figure 2A** and **Table S3**) and regarded as deNRGs. Through univariate Cox PH models, four of them were significantly associated with OS, namely, IFNA13, SLC25A5, IFNA21 and IFNA8 (**Figure 2B**, P<0.1). Three of them had a positive association with OS (HR, 95% CI of IFNA13: 1.48, 0.94–2.32; of IFNA21: 1.51, 0.98–2.32; of IFNA8: 1.46, 0.83–2.59), while SLC25A5 was negatively associated with OS (HR: 0.68, 95% CI: 0.46–1.00). Furthermore, the expression of 3504 lncRNAs differed substantially between patients and controls and were classified as delncRNAs. Through univariate Cox PH models, 422 lncRNAs remained with P<0.1 (**Figures 2C, D** and **Figure S2**).

Furthermore, Pearson’s correlation analyses were conducted, identifying 604 lncRNAs correlated with OS-related NRGs (Pa<0.05 and |r| >0.3). Of them, 31 lncRNAs were also included in the set of OS-related lncRNAs (**Figure S3**) and identified as necroptosis-related lncRNAs, which were used for the following analyses.

### Necroptosis-related lncRNA signature in IDH-wild-type GBM patients

**Figure 3:**
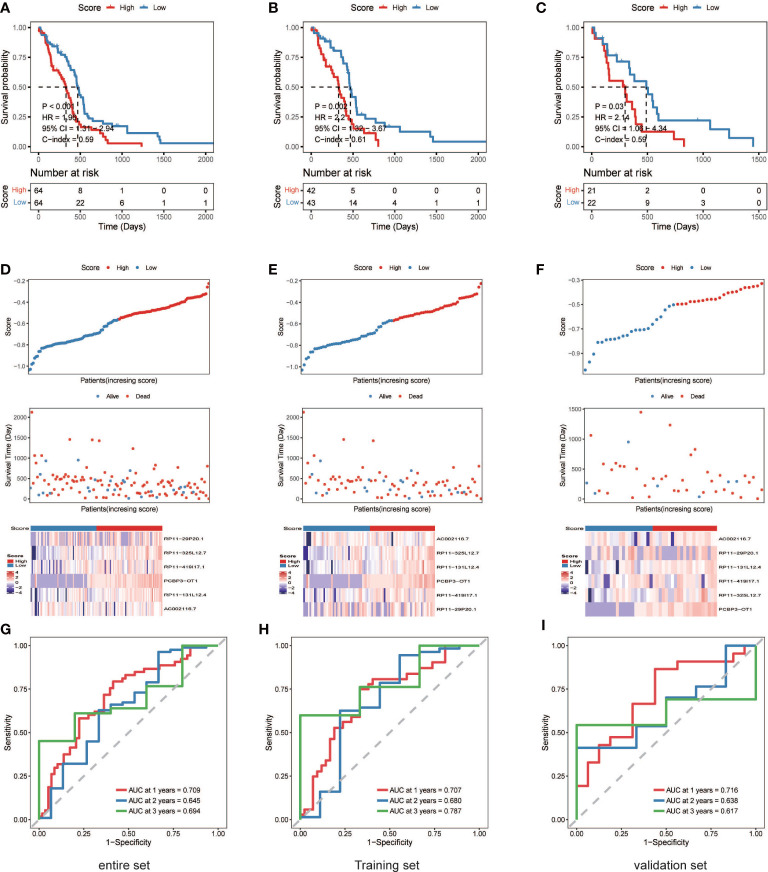
The six necroptosis-associated lncRNAs have prognostic significance. **(A)** (entire), **(B)** (training), **(C)** (validation), K-M survival curves of OS; **(D)** (entire), **(E)** (training), **(F)** (validation), Exhibition of the necroptosis-associated lncRNA model, survival time and survival status and heatmaps of the expression of 6 necroptosis-associated lncRNAs; **(G)** (entire), **(H)** (training), **(I)** (validation), ROC analysis was used to validate model performance in predicting IDH-wild-type GBM survival rates at 1, 2, and 3 years.

A nrlncRNA signature was built with a forementioned 31 nrlncRNAs through multivariate Cox PH models; after using LASSO for variable selection, when the first-rank value of log(λ) matched to the least chance of divergence, six lncRNAs with nonzero coefficients remained (**Figures 2E, F**). Based on this final model, a nrlncRNA signature for OS prediction in GBM patients was established, and each patient was assigned an RS using a linear combination of lncRNA expression weighted by their individual Cox regression coefficients, as shown below: Risk score = 0.0615×PCBP3-OT1 + 0.0367×RP11-131L12.4 + 0.0017 × RP11-419I17.1 + 0.0063 ×AC002116.7 + 5.2425×RP11-29P20.1 + 0.0276×RP11-325L12.7 (**Table S4**). There was no significant variation in RS across patients of various ages or sexes (**Figure S4**). Furthermore, the median RS was used to divide the patients into two categories, and Kaplan-Meier curves were generated, showing a positive relationship between the RS and poor OS, which was consistently observed in the training, testing and the whole datasets (**Figures 3A–F**). When examining PFS, in either of these three datasets, no meaningful correlation was discovered (**Figure S5**). Besides that, ROC analysis was used to validate model performance in predicting IDH-wild-type GBM survival rates at 1, 2, and 3 years in entire set (0.709,0.645 and 0.694), training set (0.707,0.680 and 0.787), and validation set (0.716,0.638 and 0.617) (**Figures 3G–I**).

### Construction of a prediction model for survival outcomes in patients with IDH-wild-type GBM

**Figure 4:**
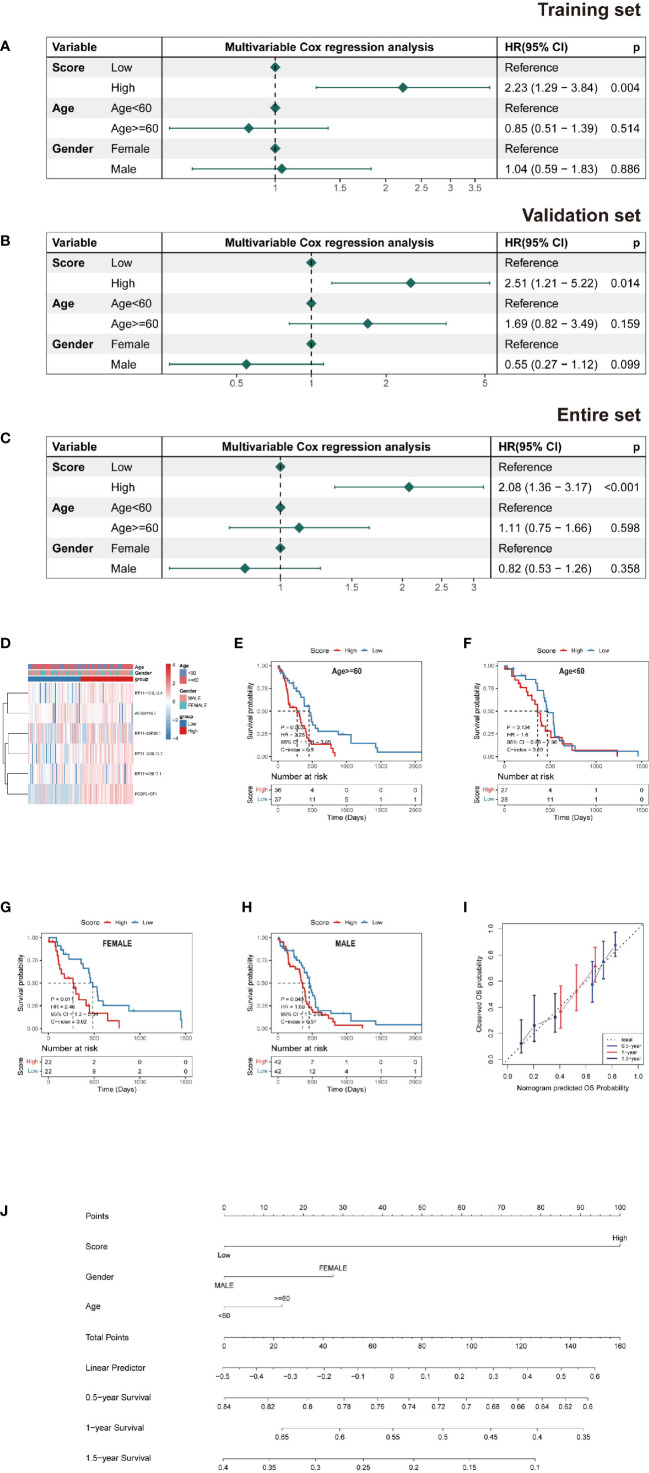
Nomogram and risk model evaluation. **(A–C)** Multivariate Cox analyses of clinical factors and risk scores with OS in the **(A)** training set, **(B)** validation set and **(C)** entire set. **(D)** Heatmap show each patient’s clinical characteristics and risk score in the whole TCGA dataset. **(E–H)** Survival analysis by subgroup. **(I)** Calibration curves for 0.5-, 1-, and 1.5-year OS. **(J)** Nomogram including tumor stage, risk score and age, to estimate 0.5-, 1-, and 1.5-year OS probabilities.

A multivariate Cox PH model was used to build a prediction model including RS, age, and gender, which revealed that RS was an independent predictor in the training, testing, and overall datasets. Patients with a high RS had a worse chance of survival than those with a low RS (**Figures 4A–H**). Based on the findings of this regression, a nomogram was created (**Figure 4J**). The nomogram is made up of nine rows, each with its own representation; the first row (points) is the point assignment for each variable. Each variable is allocated a point based on its value for an individual patient by drawing a vertical line between the exact variable value and the points line. Following that, a total point score (row 5) may be derived by adding all of the points awarded to the three variables. Drawing a vertical line between the total points and the final three rows yields the 0.5-, 1.0-, and 1.5-year survival probability. Calibration plots revealed a high degree of agreement between the predicted 0.5-, 1.0-, and 1.5-year OS and the actual OS (**Figure 4I**).

### Biological functions related to necroptosis-related lncRNAs

**Figure 5:**
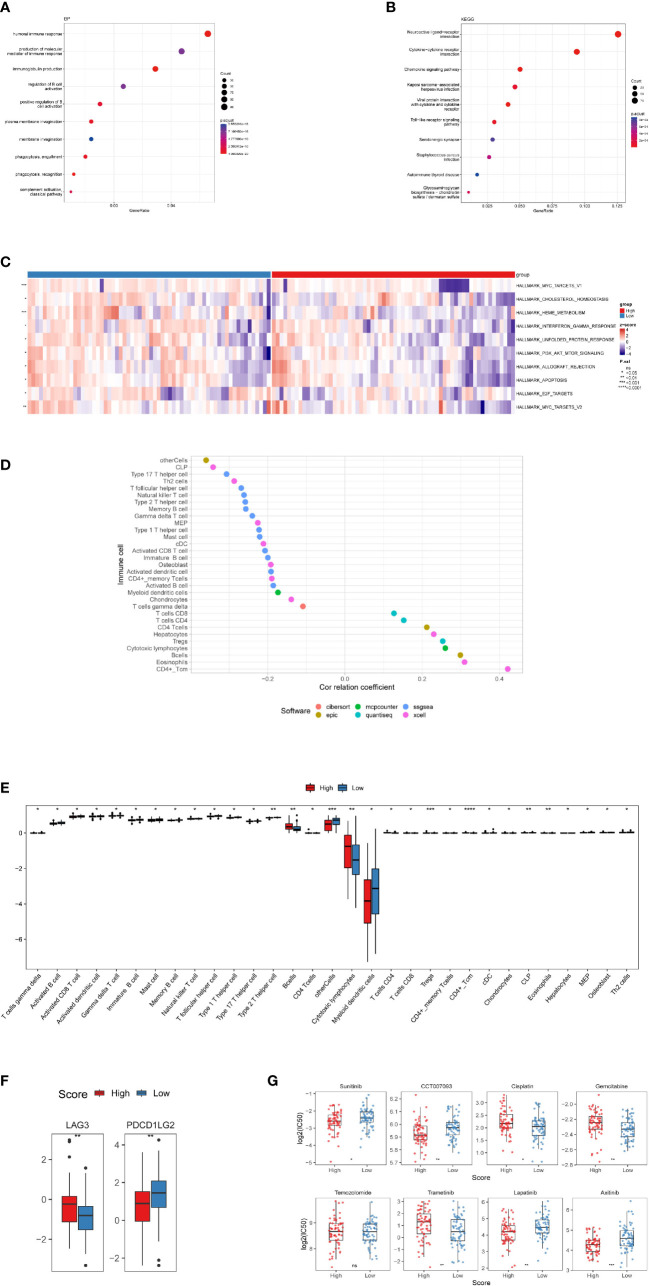
Investigation of tumor immune factors and chemotherapy. **(A)** The findings of a BP enrichment analysis of differentially expressed genes in high-risk and low-risk groups. **(B)** KEGG enrichment analysis using a bubble graph. **(C)** Gene set variation analysis (GSVA) between the high- and low-risk subgroups. **(D)** Correlation of immune cells. **(E)** A box plot depicting the differing proportions of tumor-infiltrating cells between the high-risk and low-risk groups. **(F)** LAG3 and PDCDILG2 expression levels in the high-risk and low-risk groups. **(G)** As high-risk scores were associated to the half-maximal inhibitory concentration (IC50) of chemotherapeutics, the prognostic signature was employed as an indication for chemosensitivity. (*, p<0.05; **, p<0.01; ***, p<0.001; ****, p<0.0001; ns, p>0.05).

In terms of the differentially expressed genes dictated by nrlncRNAs between the low- and high-risk groups, GSVA found possible hallmark gene sets with Pa<0.05, and the top ten are shown in **Figure 5C** and **Table S5**. Most of these pathways are related to cell survival. Additionally, functional enrichment analysis using GO keywords and KEGG pathways confirmed the link with immunity. Five of the top ten BP terms (Pa<0.05) were immunity-relevant, namely, “humoral immune response”, “production of molecular mediator of immune response”, “immunoglobulin production”, “regulation of B-cell activation” and “positive regulation of B-cell activation”. Similarly, in KEGG, out of the top ten pathways, the pathways “cytokine−cytokine receptor interaction”, “chemokine signaling pathway” and “Toll−like receptor signaling pathway” were involved in immunity (**Figures 5A, B** and **Figure S6**). Therefore, an immunity analysis was performed in the following analyses.

### Immune infiltration in IDH-wild-type GBM and its association with RS

In terms of particular immune cell type infiltration, patients with a low RS had a higher abundance of most immune cells, such as MDSCs, type 2 T helper cells and activated CD8 T cells. Moreover, the low-RS group was associated with more immune cells, including eosinophils, activated CD4 T cells and CD4 T cells (**Figures 5D, E**). There was no variation in immunological score between subgroups of individuals with high and low RSs (**Figure S7B**), and no correlation between the immune score and RS was observed (**Figure S7A**). In terms of immune checkpoint activation, two of them (PDCD1LG2 and LAG3) performed better in the low-risk and high-risk groups, respectively (**Figure 5F**).

### Clinical treatment investigation in patients with IDH-wild-type GBM

There were significant variations in IC50 values between the high- and low-risk groups for seven medications. Four of them had lower IC50 values in the high-risk group, namely, sunitinib, CCT007093, lapatinib and axitinib, while cisplatin, gemcitabine and trametinib showed higher IC50 values in the high-risk group. However, there were no variations in temozolomide levels between the two groups (**Figure 5G**).

### Knockdown of lncRNA-RP11-131L12.4 attenuates IDH-wild-type GBM cell proliferation and promotes necroptosis

**Figure 6:**
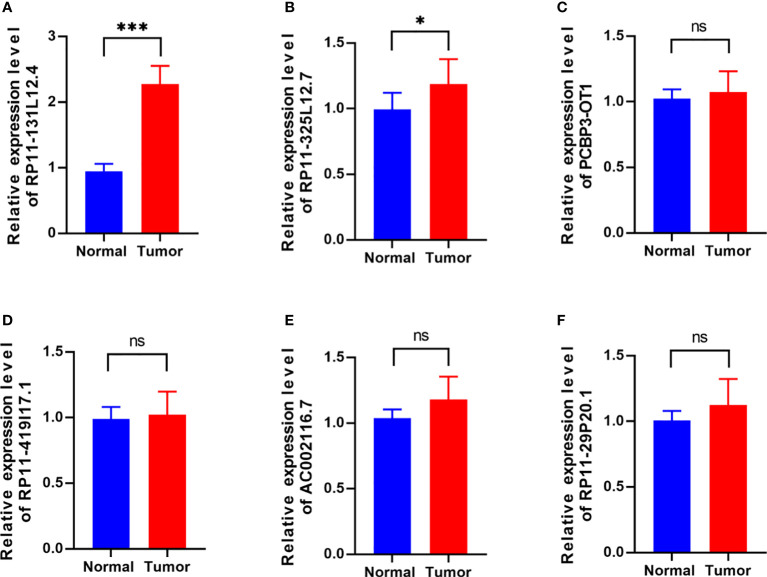
Differential expressions of six-necroptosis-associated lncRNA signature between IDH-wild-type GBM tissues and corresponding peritumor samples. **(A)** RP11-131L12.4, **(B)** RP11-325L12.7, **(C)** PCBP3-OT1, **(D)** RP11-419117.1, **(E)** AC002116.7, **(F)** RP11-29P20.1. (*, p<0.05; ***, p<0.001; ns, p>0.05).

**Figure 7:**
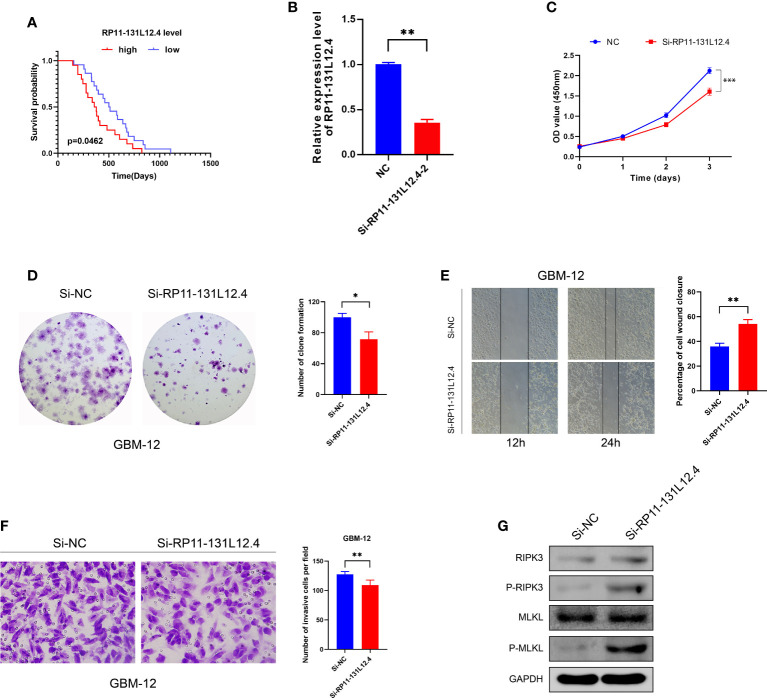
Effects of RP11-13 IL 12.4 inhibition on IDH-wild-type GBM cell proliferation, migration, invasion, and necroptosis. **(A)** Kaplan-Meier survival study for IDH-wild-type GBM patients with varying amounts of RP11-131L12.4 expressions. **(B)** RP11-131L12.4 was downregulated in IDH-wild-type GBM primary cells using siRNAs. **(C-F)** CCK-8, colony formation, wound-healing, and Transwell assays were used to assess the proliferation, migration, and invasion of IDH-wild-type GBM cells treated with siRNA targeting RP11-131L12.4. **(G)** RIPK3, P-RIPK3, MLKL, and P-MLKL were examined by Western blotting. (*, p<0.05; **, p<0.01; ***, p<0.001).

**Figure 8:**
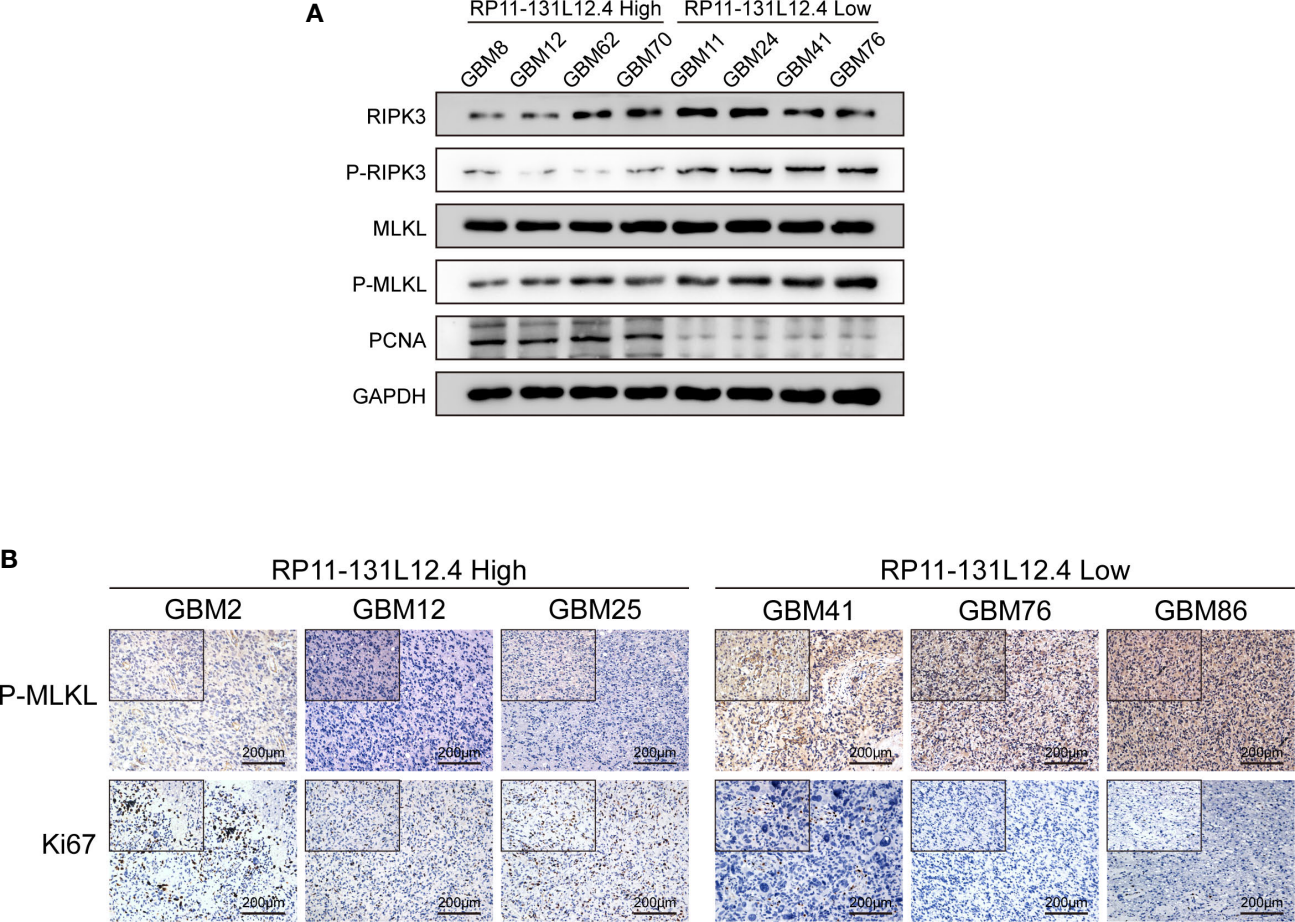
LncRNA-RP11-131L12.4 increases tumor proliferation and decreases necroptosis upon IDH-wild-type GBM tissues. **(A)** RIPK3, P-RIPK3, MLKL, P-MLKL and PCNA were examined by Western blotting in different groups. **(B)** IHC detection was used to evaluate the expression of Ki67 and P-MLKL in different groups.

To confirm our signature, we used PCR to verify the content of lncRNAs in clinical IDH-wild-type GBM tumor tissues and corresponding peritumor tissues. The expressions of lncRNA-RP11-131L12.4 and lncRNA-RP11-325L12.7 were found to be higher in tumor specimens. The statistical difference of lncRNA-RP11-131L12.4 expressions between tumor and peritumor tissues was greater (**Figure 6**). Therefore, we chose lncRNA-RP11-131L12.4 to confirm our signature. First, according to the Kaplan–Meier analysis results, increased lncRNA-RP11-131L12.4 levels predicted poor OS in GBM (**Figure 7A** and **Table S6**). In primary GBM cells, si-lncRNA-RP11-131L12.4 transfection significantly reduced lncRNA-RP11-131L12.4 expression (**Figure 7B** and **Figure S8**). The CCK-8 and colony formation tests revealed that si-lncRNA-RP11-131L12.4-transfected primary GBM cells had considerably lower colony formation than the negative control (**Figures 7C, D**). The wound-healing and transwell assays showed that silencing lncRNA-RP11-131L12.4 significantly suppressed the migration and invasion of primary IDH-wild-type GBM cells (**Figures 7E, F**). Western blot analysis illustrated that downregulation of lncRNA-RP11-131L12.4 increased P-RIPK3 and P-MLKL, indicating the potential role of lncRNA-RP11-131L12.4 in GBM cell necroptosis (**Figure 7G**). To further confirm the function, western blot analysis showed lower expression of P-RIPK3 and P-MLKL and higher expression of PCNA in GBM tissues with higher lncRNA-RP11-131L12.4 expressions (**Figure 8A**). Immunohistochemical staining showed that lncRNA-RP11-131L12.4-overexpressing GBM tissues showed higher Ki-67 expression and lower P-MLKL expression, suggesting higher proliferation but less necroptosis (**Figure 8B**).

## Discussion

Many studies have found associations between PCD-related genes and lncRNAs and GBM prognosis, which can assist predict clinical prognosis (23). Necroptosis is a kind of ACD that is involved in tumor development and suppression and may be a novel therapeutic target for GBM patients (24). According to previous studies, IDH-wild-type GBM cells are more likely to undergo necroptosis, and the degree of necroptosis is often associated with the prognosis of GBM (7). However, there is a lack of necroptosis-associated characteristics associated with tumor prognosis. Herein, the aim of this study was to identify a unique nrlncRNA signature that might be used to predict the prognosis and immune microenvironment of IDH-wild-type GBM.

We initially identified 17 NRGs using gene expression differential analysis and Cox regression to build a predictive model. Among them, IFNA13, SLC25A5, IFNA21 and IFNA8 were significantly correlated with prognosis. In fact, IFNA gene deletion has been detected in a range of cancers, and this loss is positively connected with tumor malignancy (25, 26). At the same time, the impact of tumor immunotherapy and radiotherapy is linked to the expression of IFNA genes (27, 28). SLC25A5 inhibited the MAPK signaling pathway in colon cancer, reducing cell proliferation and increasing the expression of programmed cell death-related markers (29). In our study, INFA gene expression were reduced in IDH-wild-type GBM, but SLC25A5 gene expression was enhanced. Based on differential genes, our findings revealed that 31 nrlncRNAs impacted the survival of IDH-wild-type GBM patients, and 6 of them (RP11-131L12.4, RP11-419117.1, PCBP3-OT1, AC002116.7, RP11-29P20.1, and RP11-325L12.7) were chosen to create the prognostic signature. The six-nrlncRNA signature was found to be an independent predictive predictor in patients with IDH-wild-type GBM. The nrlncRNA signature was then used to build a prediction model.

According to our study, based on prognostic features, the 1-year, 2-year, and 3-year AUC values of the RS were 0.709, 0.645 and 0.694, respectively, which suggested that the evaluation of patient prognosis based on the RS has strong efficacy. Based on Cox regression analysis, the RS was regarded an independent risk predictor and was adversely linked with the OS of IDH-wild-type GBM patients. Moreover, we developed a nomogram to predict OS using three independent parameters (risk score, age, and gender), and the same pattern was observed in calibration plots of OS at 0.5, 1.0, and 1.5 years. These findings suggest that the risk model has a good level of stability and validity for predicting the prognosis of IDH-wild-type GBM patients.

Furthermore, employing these differentially expressed necroptosis-associated lncRNAs, GO and KEGG analyses indicated that they were predominantly engaged in the MYC signaling route, PI3K-AkT-mTOR signaling circuit, E2F target signaling pathway, immune-related biological processes, and so on. The MYC gene is one of the most studied nucleoprotein oncogenes, and previous research has discovered that MYCs regulate a wide range of genes involved in cell cycle control, metabolism, and apoptosis regulation (30). Moreover, the PI3K/Akt/mTOR signaling pathway has long been recognized to increase glioma invasiveness, angiogenesis, and migration (31–33). Phosphorylation of Akt plays an important role and is regulated by molecules such as PTEN and RTK (34, 35). IDH1 influences GBM migration by regulating the PI3K/AKT/mTOR signaling pathway (33). E2F transcription factors are members of a family that play critical roles in controlling cell cycle equilibrium *via* a transcriptional axis (36). Among them, E2F1 overexpression in patient tissues is likewise associated with a poor prognosis (37, 38).

Based on the findings of functional enrichment analysis, we conducted immune analysis to determine the link between necroptosis and the immune microenvironment in IDH-wild-type GBM. According to the immune factor analysis, the low-risk group had higher immune cell infiltration, including CD8+ T cells, MDSCs, type 2 T helper cells, and other tumor-killing immune cells, whereas the high-risk group had an immunosuppressive TME. CD8+ T cells can destroy GBM cells, and greater CD8+ T cell infiltration enhances survival (39). Through the PD-1/PD-L1 immunosuppression axis, CD8+ T cells can break immunosuppression tolerance and improve immunotherapy (40). Th2 cells do not directly cause cytotoxicity, but they do facilitate it. Their effectors function by producing cytokines, such as IL-13,IL-4 and IL-5, that activate other immune cells (41–43). There were changes in the expression of immunological checkpoints between the two groups in addition to the degree of immune infiltration. Because the low-risk group had increased PDCD1LG2 activity, these individuals may react favorably to immunotherapy. Studies have shown that TIL deficiency and immune checkpoint expression deficiency are causes of tumor insensitivity to ICIs (44). The inflammatory response caused by necroptosis can change the TME and heighten the tumor response to ICIs (45).

Moreover, we studied the sensitivity of chemotherapeutic agents in different subgroups with the IC50 value. Cisplatin, gemcitabine, trametinib, and axitinib sensitivity was stronger in high-risk patients. Low-risk individuals were more sensitive to sunitinib, lapatinib, and CCT007093. However, temozolomide showed no significant difference. The drug sensitivity analysis results showed that the risk model and tumor subtypes may be used to guide treatment for IDH-wild-type GBM patients.

In addition, experiments were conducted to evaluate the functional phenotypic significance of lncRNA-RP11-131L12.4. The expression levels of six nrlncRNAs were compared between clinical IDH-wild-type GBM tumor and corresponding peritumor tissues, and we discovered that lncRNA-RP11-131L12.4 was substantially expressed in tumors and had a negative correlation with patient prognosis. *In vitro* analysis showed that inhibition of lncRNA-RP11-131L12.4 blocked proliferation, migration and invasion, and activated necroptosis in IDH-wild-type GBM primary cells by triggering P-RIPK3 and P-MKML. Moreover, immunohistochemical staining and western blotting also found that IDH-wild-type GBM tissues with high lncRNA-RP11-131L12.4 expression had stronger proliferation ability and less necroptosis. These results indicate that lncRNA-RP11-131L12.4 might be a potential necroptosis-related lncRNA in IDH-wild-type GBM.

In fact, the use of bioinformatics to find biomarkers to predict the prognosis of patients by different characteristics of tumors is very common in many types of tumors (46–48). However, due to the many influencing factors involved, it is often difficult to summarize the results with deterministic significance. Through our research methods and basic strategies, it is hoped that biomarkers based on other phenotypes can be mined. Meanwhile,the diagnosis and treatment of GBM patients in rural hospitals have encountered unique challenges due to the challenge of detection technology (49, 50). According to our results, if future studies identify the mechanism between biomarker and disease, the development of kits with easier results will be of great benefit to the treatment of GBM in rural hospitals.

## Conclusion

Our findings constructed a prognostic prediction model for necroptosis-associated lncRNAs in IDH-wild-type GBM. Moreover, the necroptosis-associated RS corresponds with the status of the TME and the expression of TILs and immunological checkpoint markers, according to our findings. Targeting necroptosis-associated lncRNAs may be another promising approach for the immunotherapy of IDH-wild-type GBM. Therefore, the mechanisms and relationships among necroptosis, lncRNAs, immunity, and IDH-wild-type GBM are worthy of further study and verification.

## Data availability statement

The original contributions presented in the study are included in the article/**Supplementary Material**. Further inquiries can be directed to the corresponding authors.

## Ethics statement

Written informed consent was obtained from the individual(s) for the publication of any potentially identifiable images or data included in this article.

## Author contributions

CS and LZ contributed to this research equally Conceptualization and study were assisted by YL, CS and SX. CS, LS, JG and SX contributed to the methodology, data analysis, visualization, and original draft writing. CS, LZ, SX, TW and CL all helped with writing and editing. LC and YL helped with financing procurement and project management. All authors contributed to the article and approved the submitted version.

## Glossary

**Table:**

ACD	(active cell death)
AUC	(areas under the ROC curve)
BP	(biological process)
CC	(cellular component)
CCK8	(Cell Counting Kit-8)
CNS	(central nervous system)
delncRNA	(differentially expressed lncRNA)
deNRG	(differentially expressed NRG)
FC	(fold change)
GBM	(glioblastoma)
GO	(Gene Ontology)
GSVA	(gene set variation analysis)
GTEx	(Genotype-Tissue Expression)
HCC	(hepatocellular carcinoma)
ICIs	(immune checkpoint inhibitors)
IC50	(half-maximal inhibitory concentration)
KEGG	(Kyoto Encyclopedia of Genes and Genomes)
LASSO	(least absolute shrinkage and selection operator)
lncRNA	(long noncoding RNA)
MF	(molecular function)
MLKL	(mixed lineage kinase domain-like)
NRGs	(necroptosis-related genes)
nrlncRNA	(necroptosis-related lncRNA)
OS	(overall survival)
PCD	(programmed cell death)
PFS	(progression-free survival)
PH	(proportional hazard)
PTEN	(phosphatase and tensin homolog deleted on chromosome ten)
P-MLKL	(phosphorylated mixed lineage kinase domain-like)
P-RIPK3	(phosphorylated receptor interacting protein kinase 3)
qRT-PCR	(quantitative reverse transcription polymerase chain reaction)
RIPK1	(receptor interacting protein kinase 1)
RIPK3	(receptor interacting protein kinase 3)
ROC	(receiver operating characteristic)
RS	(risk score)
TCGA	(The Cancer Genome Atlas)
TME	(tumor microenvironment).
