# Supplementary material for: A necroptosis-related lncRNA signature was identified to predict the prognosis and immune microenvironment of IDH-wild-type GBM
Source: Front Oncol. 2022 Dec 19;12:1024208. doi: 10.3389/fonc.2022.1024208 (PMC9806237; doi:10.3389/fonc.2022.1024208)
Supplement: Supplementary file 1 [file DataSheet_1.docx]

**Supplementary figures**


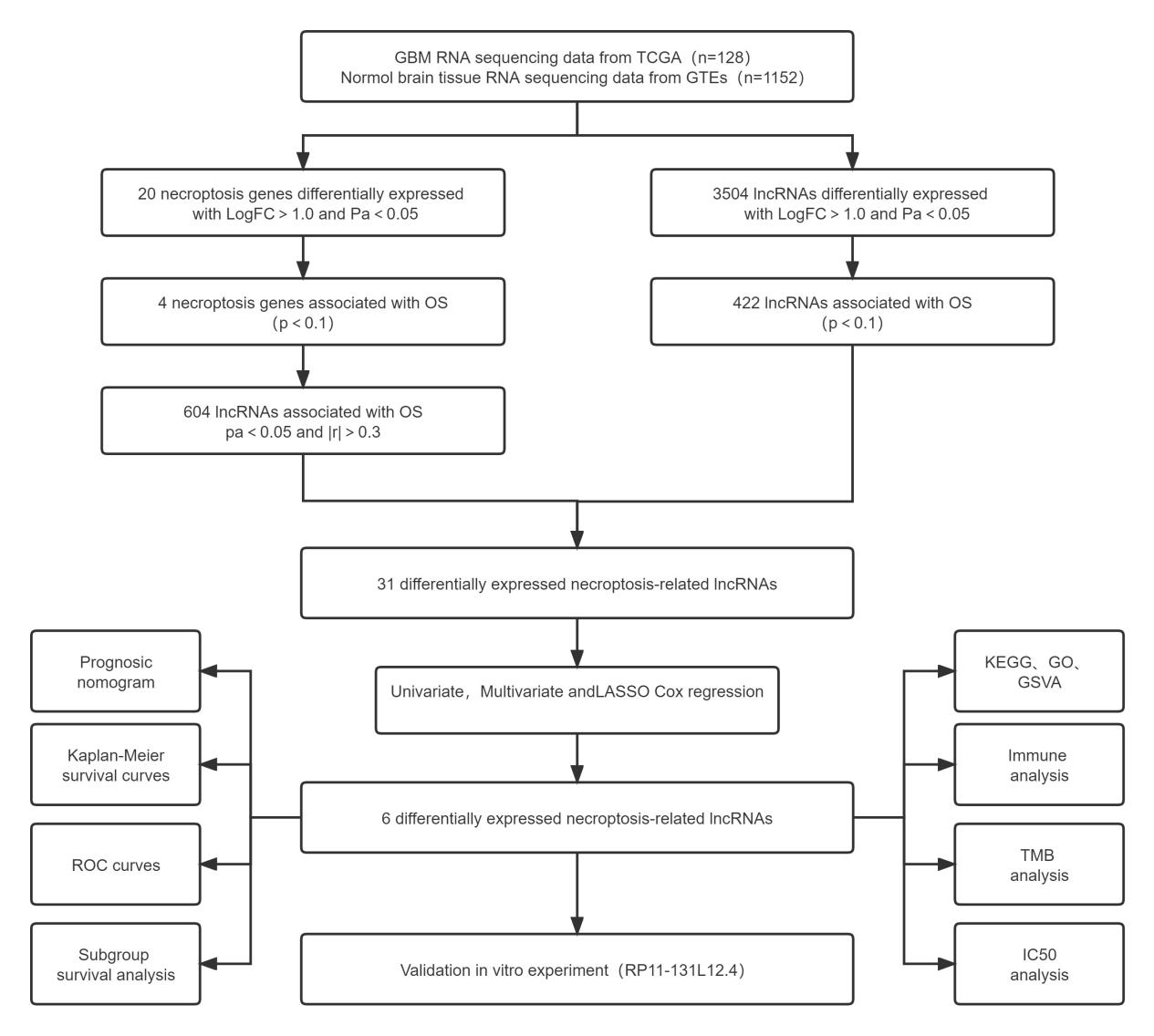


**Fig.S1** Design flow diagram for the research.


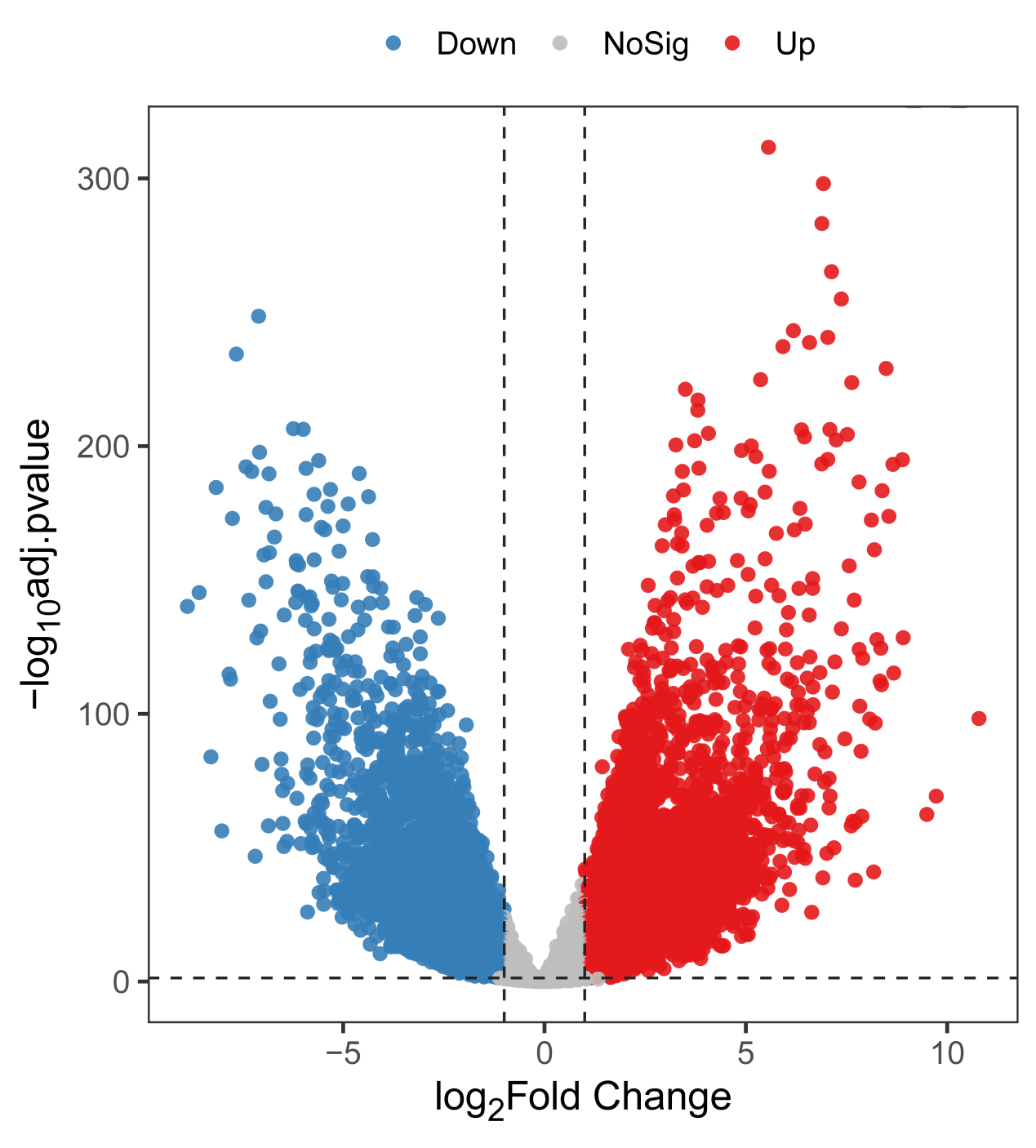


**Fig.S2.** The volcano of necroptosis-associated genes.


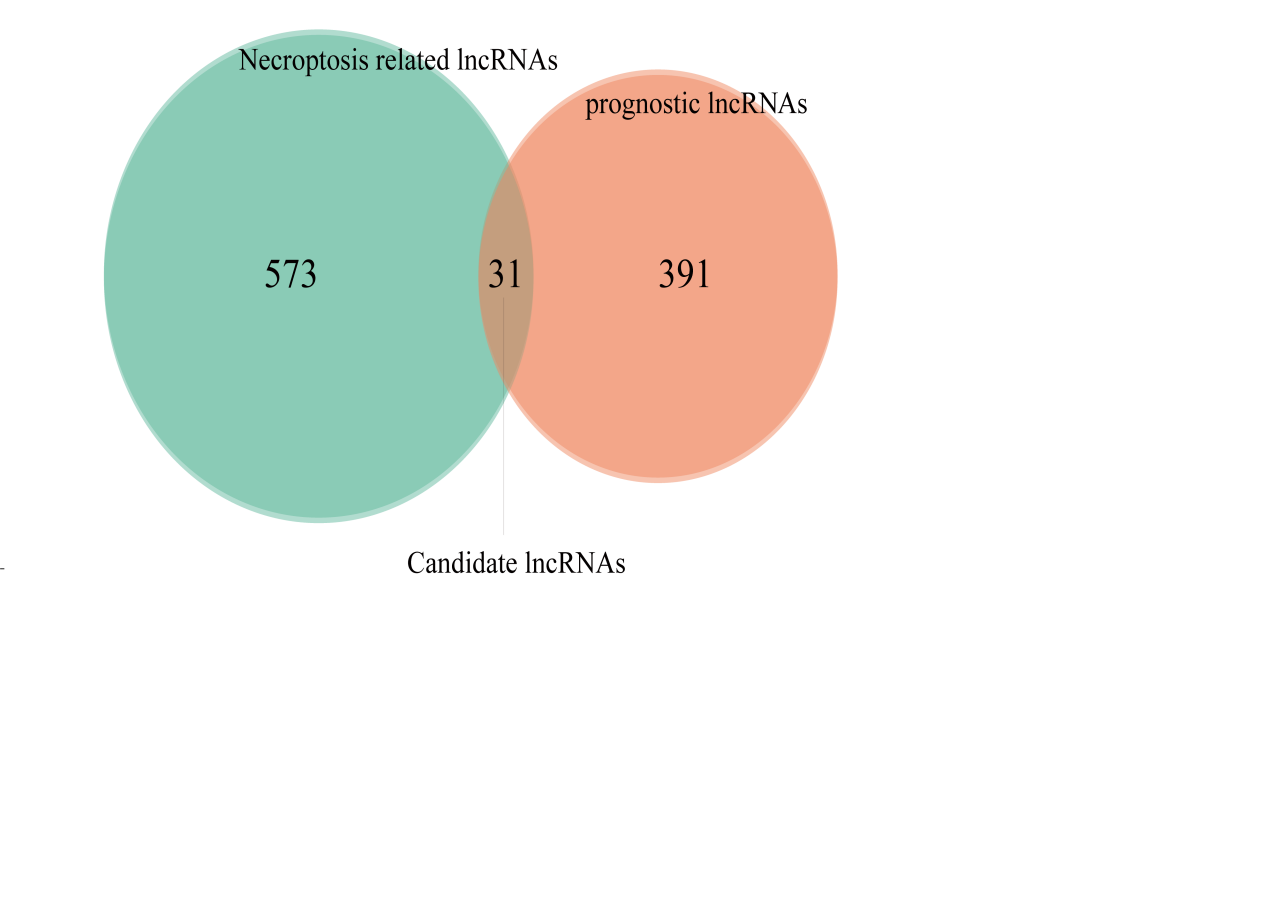


**Fig.S3** Venn diagram showed the overlap of lncRNAs between prognostic lncRNAs and necroptosis-associated lncRNAs.


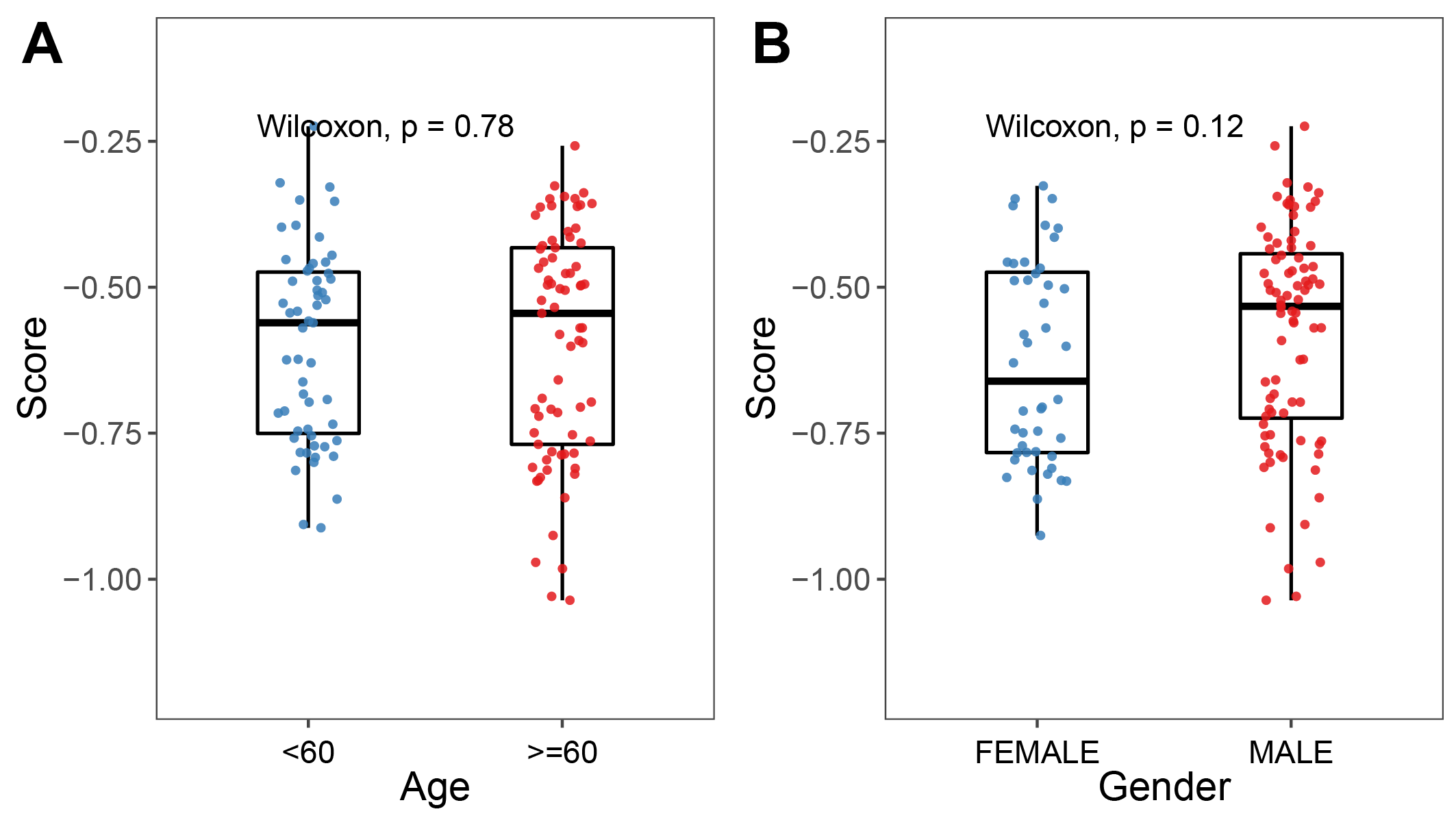


**Fig.S4** Differences in risk scores between different clinical features.


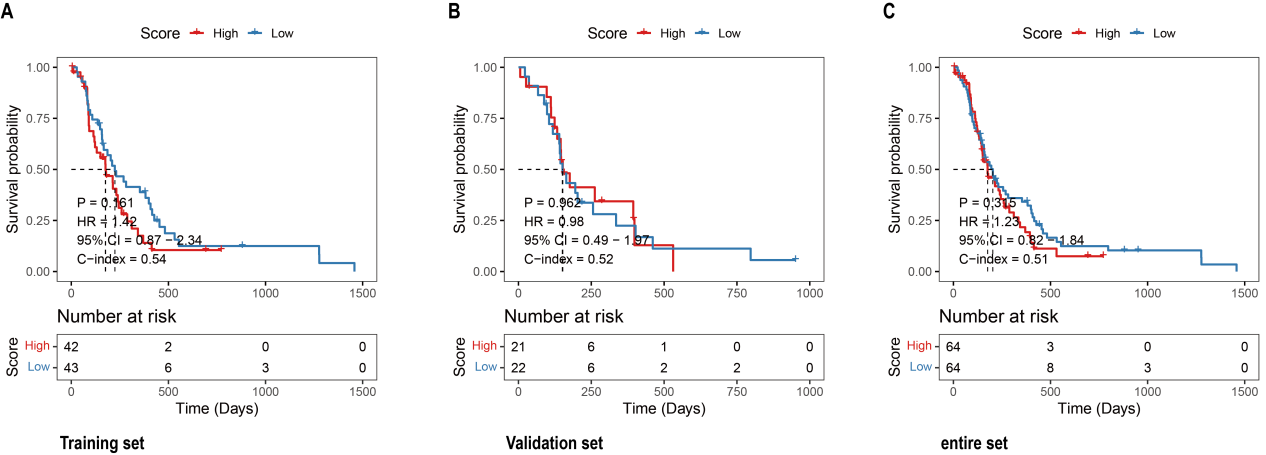


**Fig.S5** Prognosis value of the six necroptosis-associated lncRNAs model. A (entire), B (training), C (validation), (K-M) survival curves of Progression Free Survival (PFS).


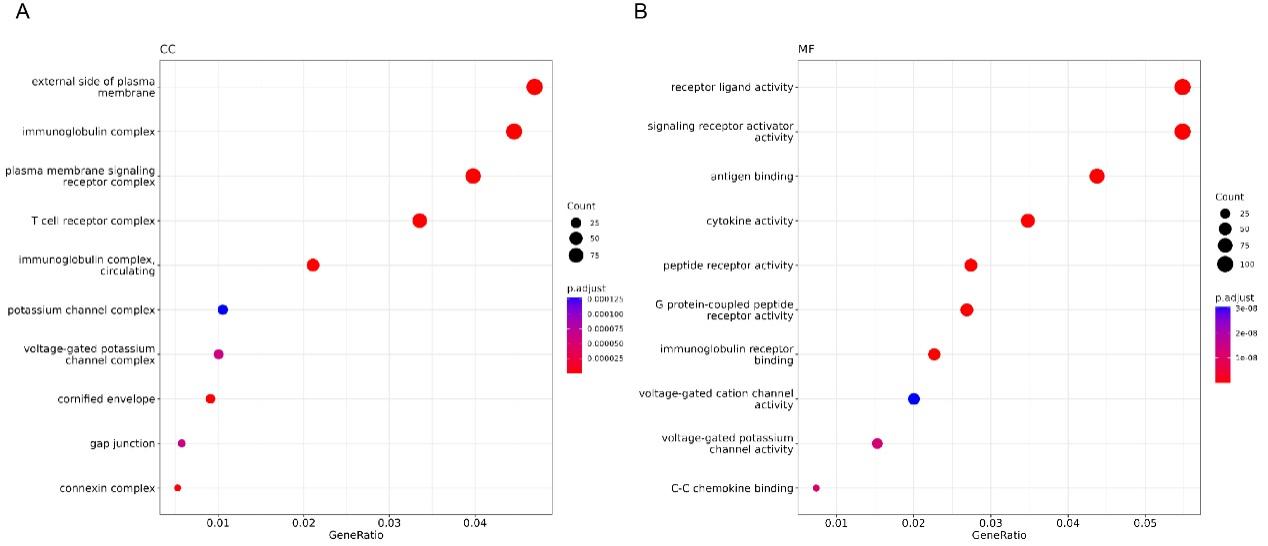


**Fig.S6** The findings of (A) Cellular Component Enrichment analysis and (B) Molecular Function of genes that differ in expression across high- and low-risk groups.


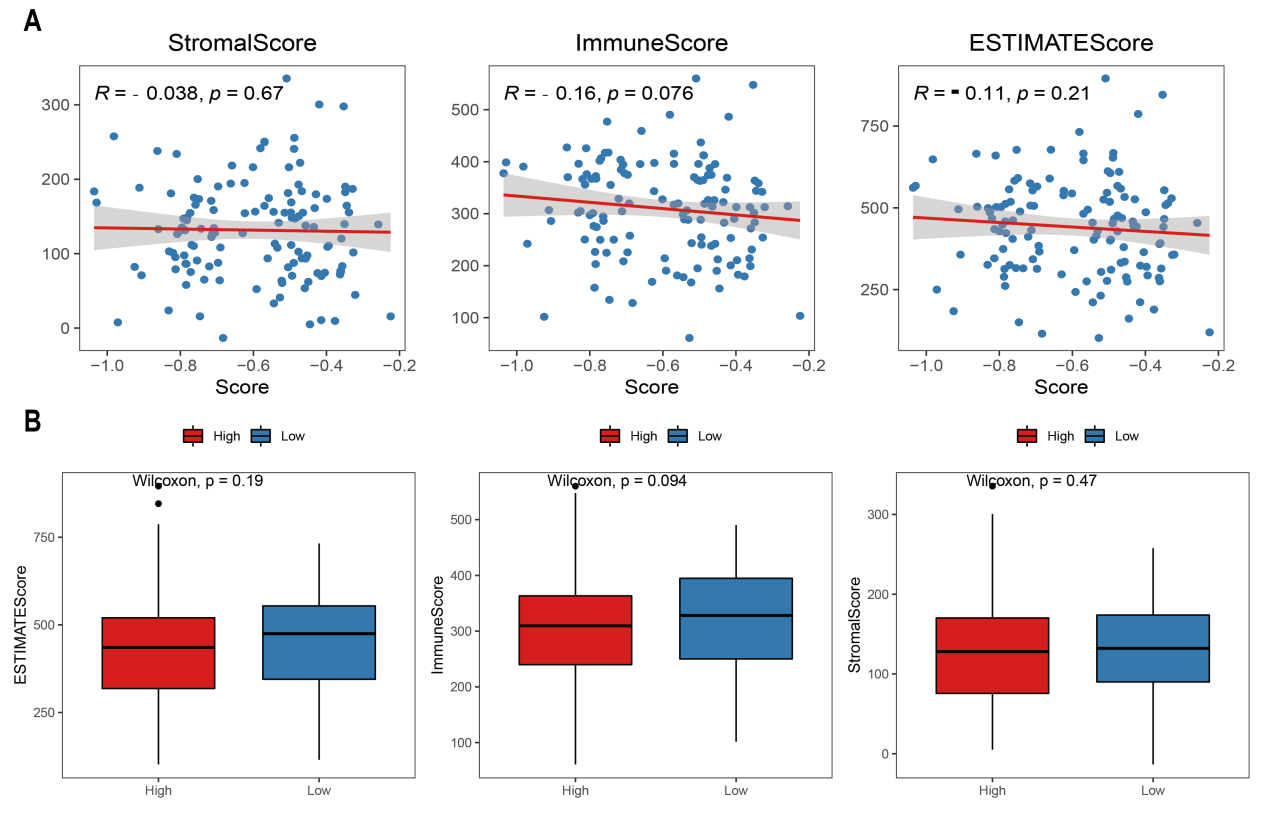


**Fig.S7** The association between risk score and tumor microenvironment score. (A, B) Stromal score, Immune score and ESTIMATE score.


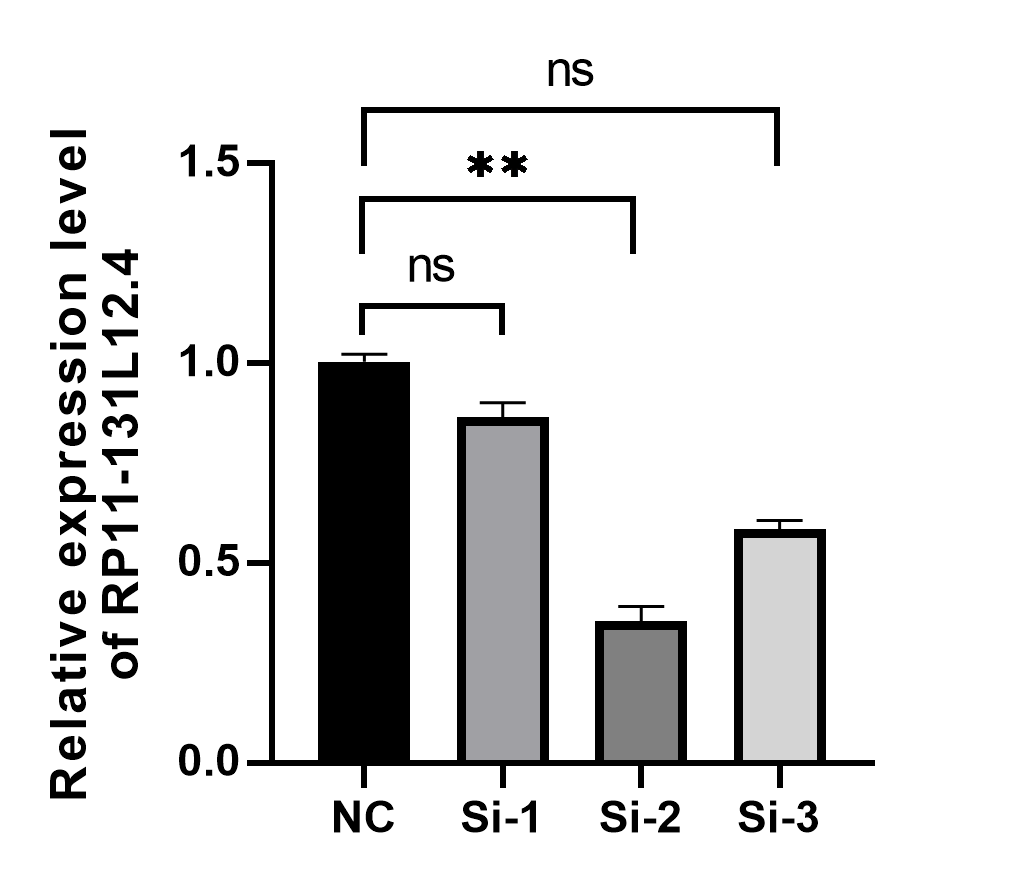


**Fig.S8** The [efficiency](javascript:;) of Si-RP11-131L12.4 in IDH wild-type GBM primary cells. (**, p < 0.01; ns, p > 0.05.)
