# Supplementary material for: A necroptosis-related lncRNA signature was identified to predict the prognosis and immune microenvironment of IDH-wild-type GBM
Source: Front Oncol. 2022 Dec 19;12:1024208. doi: 10.3389/fonc.2022.1024208 (PMC9806237; doi:10.3389/fonc.2022.1024208)
Supplement: Supplementary file 2 [file DataSheet_2.docx]

**Supplemental Materials and Methods**

**Cell culture and transfection**

The IDH wild-type GBM primary cell line (GBM #12) was obtained from Nanfang glioma sample bank of Nanfang hospital. GBM #12 cells were cultured in DMEM (Gibco, 11995065) with 10% FBS (Gibco, 16140071) and 1% penicillin/streptomycin (Gibco, 10378016) and maintained in a humidified incubator at 37°C in a 5% CO2 atmosphere. The small interference RNAs(siRNAs) targeting lncRP11-131L12.4 (targeting sequence shown in **Table S7**) were synthesized by Shanghai GenePharma Co. For cell transfection, GBM#12 cells were seeded in 6-well plates in advance and cultured overnight, at 50-60% confluence, then transfected using Lipofectamine 2000 (Thermo Fisher Scientific, 11668019) according to the manufacturer's protocol. Subsequent experiments were performed 48 h post-transfection.

**RNA isolation and qRT-PCR**

Total RNA was extracted from transfected cells or tumor samples using TRIzol® (Thermo Fisher Scientific, 15596026) according to the reagent instructions. Total RNA (1 µg) was reverse transcribed to cDNA using the reverse transcription kit (RevertAid First Strand cDNA synthesis kit) (Thermo Fisher Scientific, k1622) and genomic DNA was also removed by the gDNA Eraser from the kit. the threshold cycle (Ct) value of each sample was subsequently determined using SYBR Premix Ex Taq^™^ II Tli RNaseH Plus (TaKaRa, RR820A) on a StepOne™ Real-Time PCR system (Applied Biosystems; Thermo Fisher Scientific, Inc.). The relative expression levels of lncRNA were quantified using the 2−ΔΔCt method and normalized to GAPDH, which served as the endogenous control. All the primers used were shown in **Table S8**.

**Western blotting**

The cells were harvested and the total protein was extracted using RIPA lysis buffer (Sigma, R0278) with Combined Protease and Phosphatase Inhibitors (Thermo Fisher Scientific,78440) and protein concentrations were determined using an Enhanced BCA Protein Assay Kit (Beyotime Biotechnology, P0010). Subsequently, equal amounts of protein samples (40μg）were subjected to SDS-PAGE on 8% gels electrophoresis and electro-transferred to PVDF membranes. After blocking with 0.02 M TBS containing 5% BSA (solarbio life sciences,A8020) and 0.1% Tween-20 for 1 h at room temperature，the membranes were incubated with primary antibodies at 4 °C overnight and the HRP-linked secondary antibody for 2h at room temperature. Finally, the band intensity was quantified using the Tanon-5500 Chemiluminescent Imaging System (Tanon Science & Technology; Shanghai, China). GAPDH was used as the loading control. All the antibodies used were shown in **Table S5**.

**Cellular colony formation assays**

For colony formation assay, the transfected cells were seeded in six-well plates in a triplicate manner at a rate of 1000 cells per well. Cultured for 2 weeks, cells were fixed in 4% paraformaldehyde for 20 min and stained with 0.01% crystal violet dye for 20 min at room temperature. Then the colonies were counted by microscopy. All assays were independently repeated at least three times.

**Wound healing assay**

For wound healing assay, the transfected cells were cultured to 100% confluence in a six-well plate and then the cell monolayer was scratched with a 200μL pipette tip in a straight line to create an artificial wound. After scratch, gently wash the cell monolayer to remove detached cells and culture the remaining cells in high-sugar serum-free DMEM. Images of the same wound area were photographed at 12h and 24h under a light microscope. All assays were independently repeated at least three times.

**Cell invasive assay**

For cell invasive assay, 5 × 10^4^ transfected cells in 100μL DMEM were seeded into the matrix upper chamber (Corning, 3422),which was precoated with Matrigel ( Corning, 356234) at 37℃ overnight. In the lower chamber, 500μL of conditioned medium was added as a chemoattractant. After the cells were incubated for 8 h at 37℃ in a 5% CO2 atmosphere, the inserts were washed with PBS, and cells on the top surface of the inserts were removed with cotton swabs. Cells adhering to the lower surface were fixed with 4% paraformaldehyde, stained with 0.1% crystal violet solution, and counted under a microscope. All assays were independently repeated at least three times.

**Cell Counting Kit‑8 (CCK‑8) cell viability assay**

Cell viability was evaluated by using Enhanced Cell Counting Kit-8 (CCK8) (Beyotime, C0046) assay according to the manufacturer's protocol. Firstly, 3 x 10^3^ transfected cells per well were seeded into the 96-well plate and cultured for 1-3 days. Subsequently, 10μL of CCK8 was added into each well, and absorbance was measured at 450 nm using the BMG Microplate reader (BMG Labtech, CLARIOstar) after 2 hours of culture. All assays were independently repeated at least three times.

**Immunohistochemical staining**

The tumor samples of patients and the whole cranium from each animal were collected and fixed in 4% paraformaldehyde for 24-48 h, embedded in paraffin, cut into serial 4-µm-thick sections, and stained with hematoxylin and eosin kit (LEAGENE, DH0006-2, Beijing, China) for histological examination. The immunohistochemical staining was performed using the ZSGB-BIO PV-9000 (Beijing, China) kit according to manufacturer’s instructions. The tissue sections from paraffin-embedded human GBM specimens were stained with specific antibodies (See Supplementary table) or nonspecific IgG as a negative control.

**Statistical analysis**

The difference between the two groups was determined using the Wilcoxon test. Spearman’s test was adopted for correlation analyses. The KM analysis and Cox regression analysis were performed using the R packages “Survival” and “Survminer”. All experiments were performed in triplicate with mean and standard error of the mean reported where appropriate. Analysis of variance (ANOVA) was conducted for multi-group comparisons followed by a post-hoc Dunnett’s test (groups compared to one control group) or post-hoc Tukey’s test (to identify differences among sub-groups). Where appropriate, direct comparisons were conducted using an unpaired two-tailed Student’s t-test. A Spearman rank test was applied to verify the correlation of grading information.

**Original Images for Fig.7G**

**
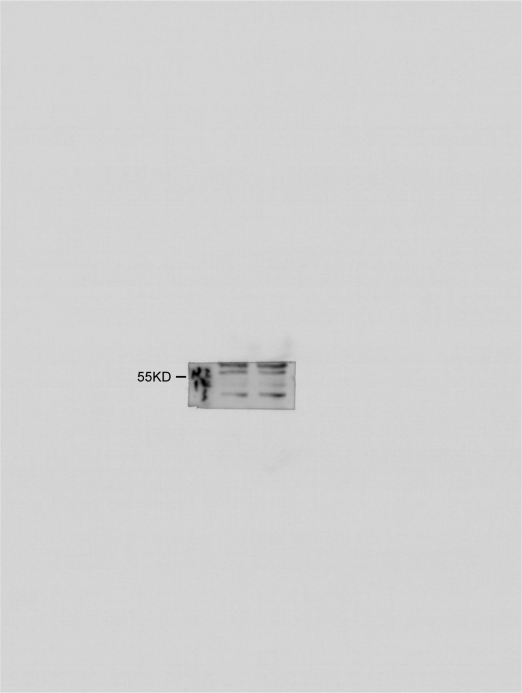
**

Original Image for Fig.7G--RIPK3


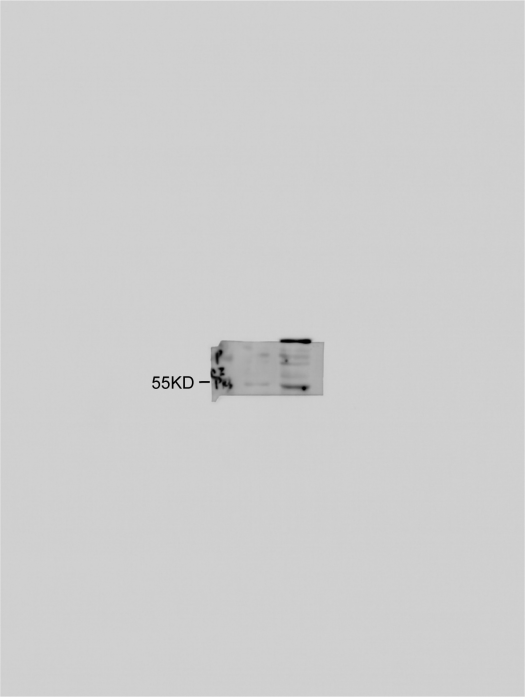


Original Image for Fig.7G--P-RIPK3


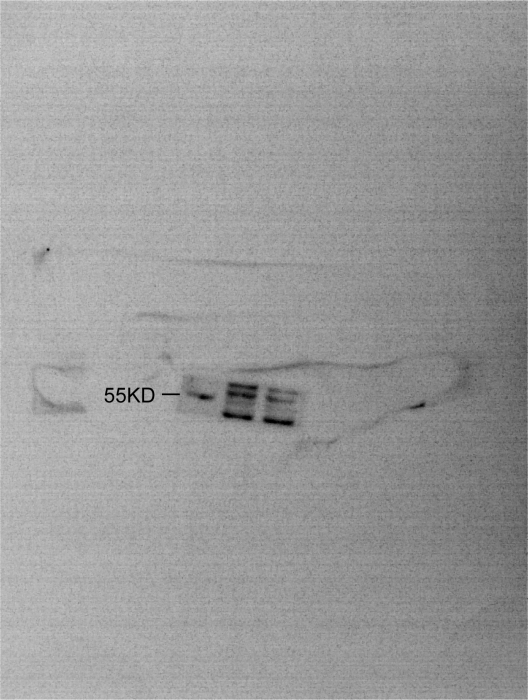


Original Image for Fig.7G--MLKL


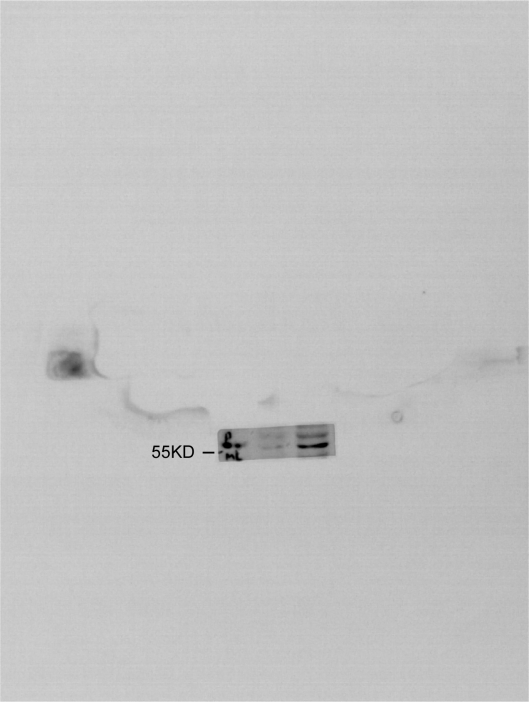


Original Image for Fig.7G--P-MLKL


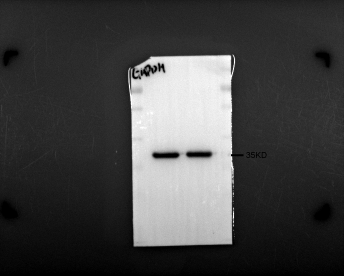


Original Image for Fig.7G--GAPDH

**Original Images for Fig.8A**





Original Image for Fig.8A—RIPK3


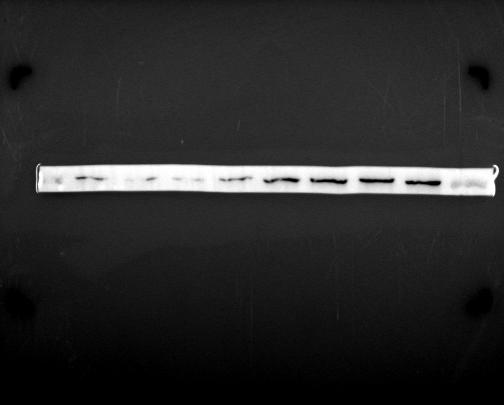


Original Image for Fig.8A—P-RIPK3





Original Image for Fig.8A—MLKL


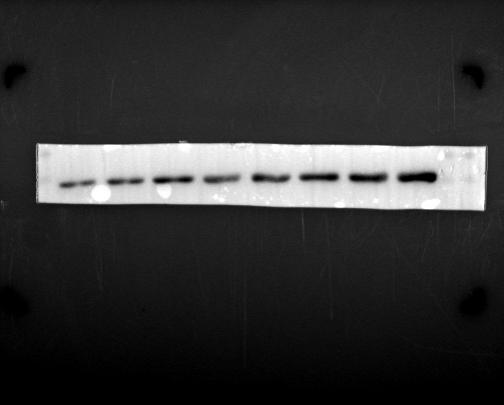


Original Image for Fig.8A—P-MLKL


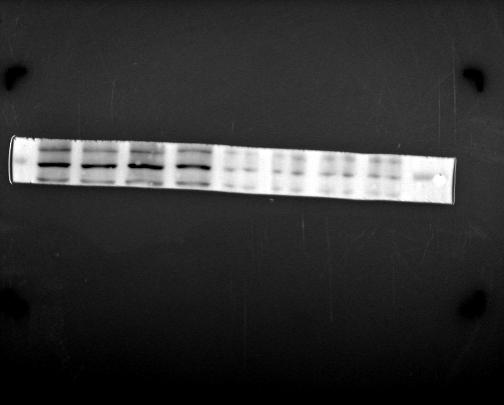


Original Image for Fig.8A—PCNA





Original Image for Fig.8A—GAPDH
